# Development of a Dendrimeric Peptide-Based Approach for the Differentiation of Animals Vaccinated with FlagT4G against Classical Swine Fever from Infected Pigs

**DOI:** 10.3390/v13101980

**Published:** 2021-10-02

**Authors:** José Alejandro Bohórquez, Sira Defaus, Rosa Rosell, Marta Pérez-Simó, Mònica Alberch, Douglas P. Gladue, Manuel V. Borca, David Andreu, Llilianne Ganges

**Affiliations:** 1 OIE Reference Laboratory for Classical Swine Fever, IRTA-CReSA, 08193 Barcelona, Spain; josealejandro.bohorquez@irta.cat (J.A.B.); rosa.rosell@irta.cat (R.R.); marta.perez@irta.cat (M.P.-S.); monica.alberch@irta.cat (M.A.); 2 Departament de Ciències Experimentals i de la Salut, Universitat Pompeu Fabra, 08003 Barcelona, Spain; sira.defaus@upf.edu (S.D.); david.andreu@upf.edu (D.A.); 3 Departament d’Acció Climàtica, Alimentació i Agenda Rural, Generalitat de Catalunya, 08007 Barcelona, Spain; 4 Plum Island Animal Disease Center, Agricultural Research Service, United States Department of Agriculture Greenport, Greenport, NY 11944, USA; douglas.gladue@usda.gov (D.P.G.); manuel.borca@usda.gov (M.V.B.)

**Keywords:** CSFV live-attenuated vaccine, serological DIVA test, FlagT4G CSFV vaccine candidate

## Abstract

Classical swine fever virus (CSFV) causes a viral disease of high epidemiological and economical significance that affects domestic and wild swine. Control of the disease in endemic countries is based on live-attenuated vaccines (LAVs) that induce an early protective immune response against highly virulent CSFV strains. The main disadvantage of these currently available LAVs is the lack of serological techniques to differentiate between vaccinated and infected animals (DIVA concept). Here, we describe the development of the FlagDIVA test, a serological diagnostic tool allowing for the differentiation between animals vaccinated with the FlagT4G candidate and those infected with CSFV field strains. The FlagDIVA test is a direct ELISA based on a dendrimeric peptide construct displaying a conserved epitope of CSFV structural protein E2. Although FlagDIVA detected anti-CSFV anti-bodies in infected animals, it did not recognize the antibody response of FlagT4G-vaccinated animals. Therefore, the FlagDIVA test constitutes a valuable accessory DIVA tool in implementing vaccination with the FlagT4G candidate.

## 1. Introduction

Classical swine fever (CSF) remains a high-impact viral disease affecting domestic and wild swine worldwide [1,2]. It is caused by the CSF virus (CSFV), a member of the *Pestivirus* genus from the *Flaviviridae* family. CSF remains endemic in Asia, areas of Central and South America, and some Eastern European countries [1]. Currently, in most endemic countries, the control of the disease relies on vaccination using live-attenuated vaccines (LAVs). This type of vaccine has proven to induce high neutralizing antibody (nAb) titers in a relatively short time after vaccination and to confer protection against highly virulent CSFV strains [3]. However, the main drawback of currently available LAVs is the lack of serological techniques to differentiate between vaccinated and infected animals (DIVA concept). This poses a limitation for their use in countries where the disease has been, or is in the process of being, eradicated, given the standards set by the World Organization for Animal Health (OIE) [4]. 

Efforts have consequently focused on the development of CSFV vaccine prototypes with DIVA capabilities, one such having been licensed in the European Union (EU), though problems with cross-reactive antibodies have been reported for its DIVA diagnostic test [5,6]. Recently, a marker LAV prototype (named FlagT4G), based on the CSFV Brescia strain, was developed [7,8]. The FlagT4G virus has a mutated T^829^AVSPTTLR^837^ epitope in the CSFV E2 glycoprotein, replacing the original T^829^SFNMDTLR^837^ sequence, as well as a synthetic Flag® (DYKDDDDK) insertion in the E1 glycoprotein [9]. However, despite having proven highly effective, inducing sterilizing immune response as early as 3 days after vaccination [8], the potential of FlagT4G still relies on an accompanying DIVA test, which has not been developed yet [1].

Considering the similarities between the FlagT4G vaccine and its parental CSFV strain, a DIVA test should ideally require antigen presentation based on the specific mutations of the E2 glycoprotein, rather than on its entirety. An attractive approach in this regard is a peptide construction where the epitope of interest is displayed as multiple copies in a branched (dendrimer) fashion [10]. This type of construct favors the recognition of epitopes by the immune system, is versatile in that it allows the display of several epitopes on a single molecular platform, and is readily accessible by synthetic technologies [10,11]. Peptide dendrimers have found practical application as drug carriers and vaccines, including a promising candidate against the foot-and-mouth disease virus [12,13]. They have also been employed to increase the sensitivity of ELISA [14,15,16].

In the CSFV field, dendrimeric peptides have provided a tool to study the ability of CSFV B and T-cell epitopes to induce efficient immune responses in combination with epitopes from other animal health pathogens [17,18,19]. In this context, the aim of the present work was to develop a dendrimeric construct harboring an epitope combination that allows for the differentiation between animals vaccinated with FlagT4G and CSFV-infected pigs using a serological test.

## 2. Materials and Methods

### 2.1. Cells and Viruses

The porcine kidney cell lines PK-15 (ATCC-CCL-33) and SK-6 were used. Cells were grown in Eagle’s minimum essential medium supplemented with 5% fetal bovine serum (FBS). Immune peroxidase monolayer assay (IPMA) was employed for viral replication monitoring using a swine polyclonal *Pestivirus* antibody [20]. Viral titers were determined by end-point dilution, calculated following standard statistical methods [21]. The CSFV Alfort/187 strain, kindly provided by the CSFV EU Reference Laboratory (Hannover, Germany), and the FlagT4G vaccine virus were used. 

### 2.2. Synthesis of Dendrimeric Peptides

**Figure 1:**
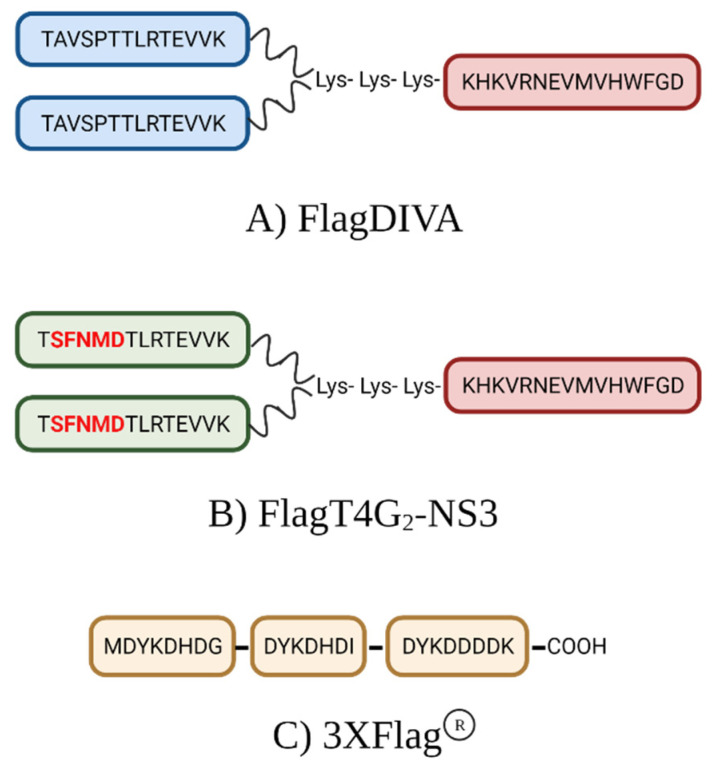
Peptides used as a coating antigen for ELISA. Serum samples from vaccinated pigs were evaluated either by the FlagDIVA (**A**), FlagT4G_2_-NS3 (**B**), or Flag® (**C**) ELISA. Red bold letters in peptide B indicate the mutation introduced in the E2 glycoprotein of the FlagT4G vaccine. Box colors indicate the different epitope peptides: blue (original CSFV E2 epitope), green (FlagT4G modified E2 epitope), red (NS3 immunogen), or yellow (commercial 3× Flag). In constructs A and B, thiol-maleimide linkages between the extra C-terminal Cys and both maleimido-derivatized α- and ε- amino ends of the branched Lys core are simplified by wavy lines. Image created using biorender.com (accessed on 22 September 2021).

Two dendrimeric peptides were synthesized by thiol-maleimide ligation using pre-purified precursors prepared by solid-phase peptide synthesis, based on a previously described methodology [12,19]. The first construct (FlagDIVA) contained two copies of the wild-type CSFV-E2 epitope (T^829^AVSPTTLRTEVVK^842^) linked to a single copy of an NS3 epitope (K^1446^HKVRNEVMVHWFGD^1460^) proven to enhance immune system recognition (Figure 1A). In the second construct (FlagT4G_2_-NS3) the two copies of the wild-type E2 epitope were replaced by the FlagT4G (T^829^SFNMDTLRTEVVK^842^) sequence and linked to the same NS3 epitope (Figure 1B). To effect the ligation, each E2 epitope was C-terminally elongated with a Cys residue, and the NS3 epitope was N-terminally elongated with two Lys plus another Lys with both amino functions modified by maleimide groups securing the branched arrangement. Dendrimeric peptides were purified by preparative reverse-phase HPLC and characterized by ESI mass spectrometry.

### 2.3. Experimental Vaccination

Six-week-old Landrace x Large white (*n* = 15) piglets were introduced into the animal biosafety level 3 facilities (aBSL3) at IRTA-CReSA in Barcelona, Spain. All the pigs had tested negative for antibodies against pestiviruses prior to entering the aBSL3. After a 5-day adaptation period, all the pigs were vaccinated with 10^5^ TCID_50_ CSFV FlagT4G virus via intramuscular injection in the right neck. Serum samples were collected from all pigs at 6, 13, 20, and 28 days post vaccination (dpv) and a boost immunization with the same vaccine dose was carried out at 18 dpv. All animals were euthanized at 28 dpv by pentobarbital overdose (60–100 mg/kilogram of weight), administered via the vena cava, in accordance with accepted methods included in European Directive 2010/63/EU. The experiment was approved by the Ethical Committee of the Generalitat de Catalunya, Spain, under the approval code 10630, on 18 December 2019.

### 2.4. Neutralizing and CSFV E2-Specific Antibody Detection

CSFV E2-specific antibodies were determined in all serum samples from the FlagT4G-vaccinated animals using a commercial CSFV ELISA (IDEXX Laboratories, Liebfeld, Switzerland). Following the manufacturer’s instructions, blocking percentage values above 40% were considered positive, between 30–40% doubtful, and below 30% negative. Serum samples were also tested for CSFV neutralizing antibodies with a neutralization peroxidase-linked assay (NPLA) [22]. Titers were expressed as the reciprocal dilution of serum that neutralized 100 TCID_50_ of the Alfort/187 strain in 50% of cell culture replicates.

### 2.5. Determination of Humoral Response Elicited by FlagT4G Using Dendrimeric Peptides and Flag® Peptide ELISA

Three different peptides were used as a coating antigen in an ELISA in order to evaluate the humoral response in serum samples from the FlagT4G-vaccinated pigs (Figure 1): FlagDIVA, FlagT4G_2_-NS3, and Flag^®^ (3× FLAG peptide, GLPBIO, Montclair, CA, USA). All serum samples from vaccinated animals were evaluated by the FlagDIVA and Flag^®^ ELISA tests, whereas samples at 28 dpv were only tested by FlagT4G_2_-NS3. A previously established ELISA protocol was performed [17,18]. Briefly, 50 µL/well of a solution containing 40 μg/mL of dendrimer or peptide diluted in sodium carbonate–bicarbonate buffer (0.05 M NaHCO_3_, 0.05 M Na_2_CO_3_, pH 9.4) were coated overnight at 4 °C on high-binding Costar 3590 plates (Corning, New York, NY, USA). Free active sites were blocked using 0.5% bovine serum albumin (BSA) in PBS for 1 h (blocking buffer). Each serum sample was pre-diluted in blocking buffer (1:25), and 50 μL/well were plated by duplicate and incubated at 37 °C for 1 h. Afterwards, 50 μL/well of anti-swine IgG peroxidase conjugate (Merck, Darmstadt, Germany) diluted 1:20,000 in the same buffer used for blocking active sites was added and plates were incubated at 37 °C for 1 h. The amount of coupled conjugate was determined by incubation with 50 μL/well of soluble 3,3′,5,5′-tetramethylbenzidine (Calbiochem, San Diego, CA, USA) for 10 min at room temperature. Finally, the reaction was stopped with 50 μL/well of 1N H_2_SO_4_ and the absorbance was determined at 450 nm. Optical density (O.D.) values above 0.4 were considered positive in the dendrimer ELISA tests.

### 2.6. CSFV Humoral Response Detection by Dendrimeric Peptides

The capacity of the FlagDIVA dendrimeric peptide to detect humoral response against CSFV was further assessed using a panel comprising 217 serum samples from the OIE CSF reference laboratory at IRTA-CReSA. The panel included 40 samples from uninfected pigs as well as 177 samples from pigs infected with different CSFV strains, all of which were previously tested for antibodies against CSFV by a commercial ELISA. In addition, in order to evaluate the capacity of FlagDIVA to differentiate antibodies against CSFV from those elicited by the recently discovered ovine pestivirus (OVPV) [23], samples from OVPV-infected pigs (*n* = 21) were tested by the FlagDIVA assay. These samples had been collected in a previous experimental infection and corresponded with animals that were positive by the commercial CSFV E2 ELISA [24].

## 3. Results

### 3.1. The Antibody Response Elicited by the FlagT4G Vaccine against CSFV Is Not Detected by the FlagDIVA Peptide 

**Figure 2:**
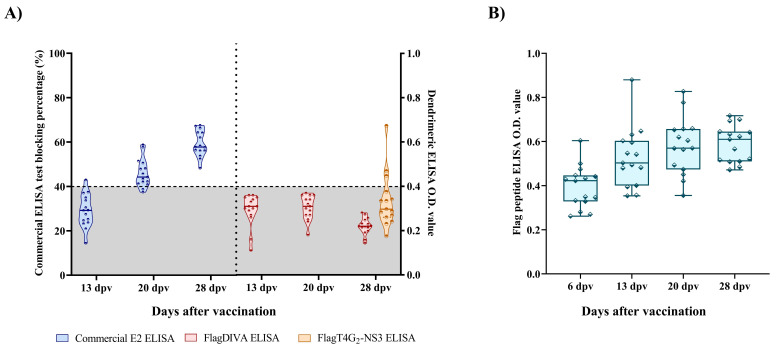
Humoral response elicited by the FlagT4G vaccine. (**A**) Antibodies against the E2 glycoprotein were evaluated by the commercial ELISA (blue shapes and dots); results are expressed as a blocking percentage value (left Y-axis). Antibody detection was also carried out using the FlagDIVA (red shapes and dots) and FlagT4G2-NS3 (tallow shape and dots) dendrimeric peptides and is expressed as an O.D. value (right Y-axis). For both assays, values corresponding to negative samples are shown in the shaded area below the horizontal dotted line (40% blocking percentage for the commercial ELISA and 0.4 O.D. for the dendrimeric peptide tests). Lines within the boxes indicate the mean value for each assay. (**B**) Humoral response detected by the Flag® peptide. Sera from the vaccinated animals was evaluated at different timepoints after vaccination; results are expressed as an O.D. value. Mean values (line within the box) are shown for each timepoint.

Specific anti-E2 antibody response was detected in one animal vaccinated with the FlagT4G virus starting at 13 dpv, using the commercial anti-E2 ELISA. Afterwards, humoral response against the E2 glycoprotein was detected in 13 out of the 15 vaccinated animals at 20 dpv and in all the vaccinated animals at 28 dpv (Figure 2A). In the Flag^®^ peptide ELISA test, O.D. values between 0.26 and 0.62 were detected in the vaccinated animals at 6 dpv. The anti-Flag™ antibody response was increased on subsequent timepoints, with mean O.D. values of 0.5, 0.57, and 0.61 at 13, 20, and 28 dpv, respectively. Conversely, no antibody response was detected by the FlagDIVA ELISA in samples from the vaccinated animals at any timepoint, whereas three animals were positive by the FlagT4G_2_-NS3 assay at 28 dpv (Figure 2B).

In the NPLA test, neutralizing antibodies were detected in 13 out of 15 pigs at 13 dpv, with titers ranging from 1:10 to 1:80. After this timepoint, neutralizing antibodies were detected in all pigs and titers increased, being as high as 1:160 and 1:640 at 20 dpv and 28 dpv, respectively.

### 3.2. Comparative Performance between the FlagDIVA ELISA and the Commercial ELISA in the Detection of e2-Csfv Antibody Response

**Figure 3:**
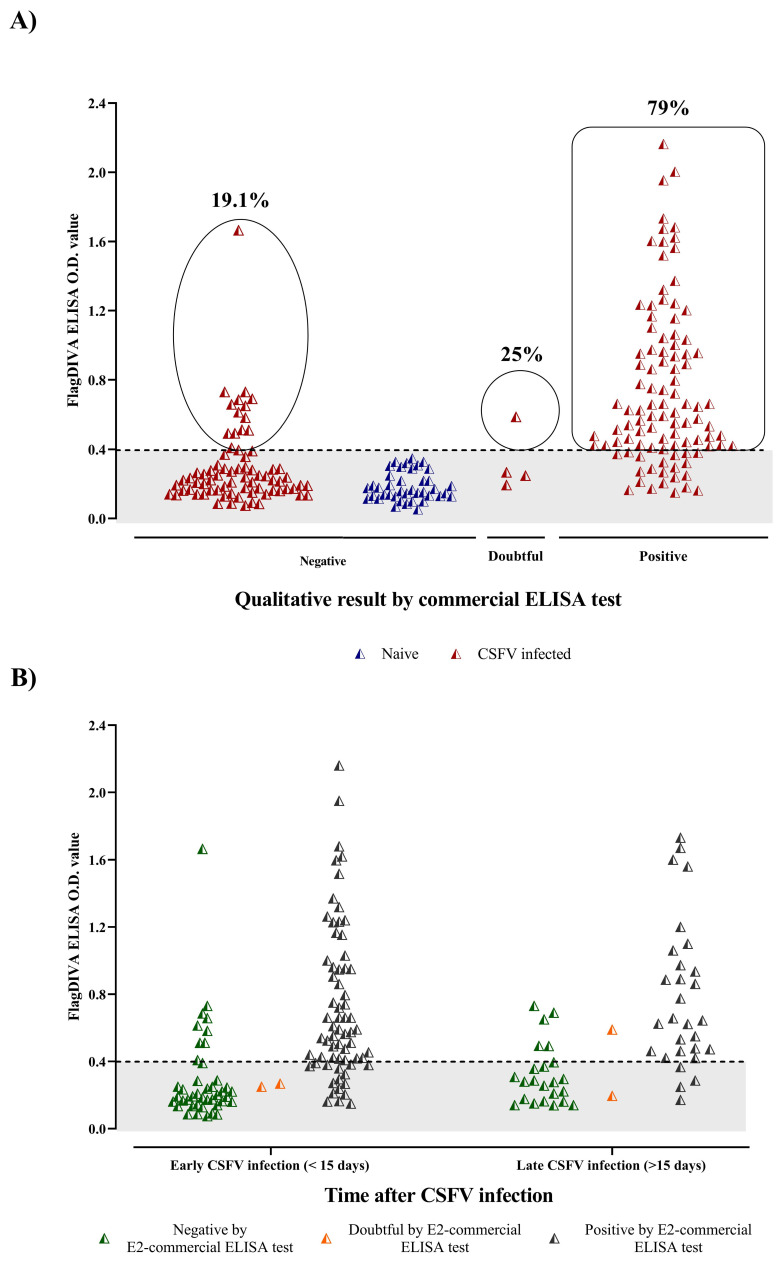
Detection of antibodies against CSFV by the FlagDIVA dendrimeric peptide. (**A**) Serum samples from naïve pigs (blue triangles) as well as animals infected with CSFV strains of different genotypes (red triangles) were evaluated by the FlagDIVA and commercial ELISA tests. Results are shown in comparison with qualitative results from the commercial assay (X-axis). (**B**) Samples from CSFV-infected animals that were either negative (green triangles), doubtful (orange triangles), or positive (gray triangles) by the commercial ELISA were subsequently compared to their results in the FlagDIVA assay according to their time after infection (X-axis). Quantitative results for the FlagDIVA assay are shown as an O.D. value (Y-axis) in both panels, with their respective threshold (dotted line).

All serum samples from uninfected animals were negative by both the commercial and dendrimeric peptide ELISA. Interestingly, 14 samples from CSFV-infected animals that had been negative for E2-CSFV antibodies by the commercial ELISA were determined to be positive by the FlagDIVA ELISA. Additionally, the dendrimeric peptide assay was able to detect humoral response in one serum sample from two infected pigs that had been characterized as doubtful by the commercial ELISA (Figure 3).

On the other hand, 79 out of the 100 samples that were positive by the commercial ELISA were also positive for CSFV antibodies by the FlagDIVA assay (Figure 3A). The majority of the remaining samples (17 out of 21) corresponded to pigs within the first 15 days of CSFV infection (Figure 3B).

In addition, all serum samples from OVPV-infected pigs, which were positive in the commercial CSFV E2 ELISA, were negative by the FlagDIVA ELISA (data not shown).

## 4. Discussion

The control and eradication of CSF at a global level continues to be a challenge in animal health [1,2,25]. Vaccination remains the most feasible strategy to achieve this goal [3,26,27]. However, eradication policies have not always been based on vaccination, but rather on strict serological surveillance and stamping out, which has proved costly and inadequate from an ethical and animal welfare standpoint [28,29]. Currently, the use of vaccines against CSFV is not permitted in countries where the disease has been eradicated, since the available vaccines do not fulfill the DIVA concept and therefore are incompatible with the serological surveillance carried out in those countries. 

Efforts have been focused on the development of vaccines that can induce antibody responses that can be differentiated from those induced by a natural CSFV infection [1,3]. However, the development of diagnostic tests able to differentiate the vaccine-induced immune response has remained an unsolved challenge.

The FlagT4G vaccine candidate has proven to induce efficient immunity against highly virulent CSFV challenge as early 3 days after vaccination [8]. In the present study, animals vaccinated with FlagT4G generated antibody response against CSFV as early as two weeks after vaccination. Furthermore, the neutralizing antibody titers induced by the vaccine in these pigs were well beyond the established threshold for protection [17,30] However, despite inducing rapid and effective immune response, the FlagT4G vaccine still lacks a reliable DIVA diagnostic technique [1]. In the present work, a serological tool (FlagDIVA test) to differentiate animals vaccinated with FlagT4G from those infected with CSFV field strains was developed. 

This diagnostic strategy is based on the negative differentiation of the vaccinated animals through the use of a dendrimeric peptide as an ELISA capture antigen. The wild-type epitope (TAVSPTTLRTEVVK) used in multimeric form in the FlagDIVA peptide and reported as highly immunogenic has been mutated in the FlagT4G vaccine, as well as in other attempts to generate CSFV DIVA vaccines [7,31,32]. The mutations introduced in the FlagT4G vaccine led to a failure in the recognition of the wild-type epitope by the antibodies generated in the vaccinated pigs, even after 28 dpv, when efficient induction of immune response by the FlagT4G was able to be easily detected in those pigs. Nonetheless, the FlagDIVA ELISA was able to detect antibody response in serum samples from animals infected with CSFV field strains. Notably, 19.1% of samples from CSFV-infected pigs that were negative by the commercial ELISA were positive by the FlagDIVA assay. Some of these samples were positive by the NPLA test with titers between 1:5 and 1:10. 

The detection of the antibodies generated after infection with field strains was likely aided by the multimerization of the wild-type epitope in the FlagDIVA peptide. Previous reports have shown antigen multimerization to enhance immune recognition [10]. Additionally, the versatility provided by the dendrimeric constructs allows for the inclusion of another CSFV epitope in the same molecule, which may further increase its detection by antibodies of CSFV-infected animals.

Even so, detection of CSFV antibodies generated by field strains was not flawless, since 18 samples from CSFV-infected pigs, positive by the commercial ELISA, were negative by the FlagDIVA assay. However, it is important to remark that most of those samples were collected at short post-infection times (less than 15 days, Table S1). Further studies will be required to improve early detection of CSFV antibodies with the dendrimeric approach. In addition, the capacity of the FlagDIVA test to detect antibodies against CSFV in pigs vaccinated with FlagT4G following infection with field CSFV strains will also be a subject of study. The highly conserved TAVSPTTLRTEVVK motif across CSFV field isolates would be a good target to provide high stability for antibody detection. Moreover, taking into account the advantages of the dendrimeric peptides, in terms of versatility to include different epitopes in the same construct [10,13], the addition of epitope sequences that reflect the variability found in field conditions for the NS3 epitope would also increase the sensitivity of the assay.

The fact that serum samples from OVPV-infected pigs were negative by the FlagDIVA assay supports the specificity of this ELISA, considering the high genetic and antigenic similarities between this virus and CSFV [24,33].

Great efforts have been invested in creating a DIVA vaccine against CSFV with a sensitive diagnostic test [3]. This has led to promising candidates like CP7_E2alf, which is able to confer rapid and efficient immune response and has been granted marketing authorization in the European Union [5,34]. Nonetheless, the DIVA diagnostic test used for this vaccine, based on E^rns^, another CSFV structural protein, has been shown to detect antibody responses in pigs after infection by other pestiviruses, posing an important limitation for this assay as a DIVA indicator [6]. In addition, the antibody response generated against E^rns^ is slow in comparison to that against E2, and not always activated by low-virulence CSFV strains [35].

Other approaches to generate a marker vaccine have focused on the C-strain, due to its efficacy in generating a protective response [1,3]. Thus, a modified C-strain vaccine, named C-DIVA and carrying mutations in the same epitope as the FlagT4G vaccine, has proven to induce clinically protective immunity as early as 7 days after single vaccination [29,36]. Moreover, the DIVA diagnostic test used for this vaccine candidate is based on a modification of a well-established commercial ELISA, seemingly without affecting sensitivity and specificity [37]. The main drawback of this candidate appears to be its failure to provide protection, as viral RNA was detected in leukocytes and body secretions of vaccinated pigs during the first week after challenge, suggesting viremia and viral excretion, respectively [31]. Moreover, the use of this C-strain vaccine for over 50 years, sometimes under suboptimal conditions, has promoted viral evolution, leading to viral attenuation and the possible emergence of vaccine escape variants in the field [25,38]. 

The results of the present study support the role of the FlagT4G vaccine as a tool for CSFV eradication, accompanied by the FlagDIVA assay as its diagnostic test. FlagDIVA might be used in combination with the commercial E2-based ELISA, rather than replacing it, thus improving overall sensitivity. Further studies are under way to fully assess the capacity of FlagDIVA to differentiate the immune response elicited by CSFV from other pestiviruses. 

## 5. Patents

The work reported in this manuscript has resulted in the application for a European patent (application number: EP2138539.1) titled “Peptide-based assay to differentiate animals infected with CSFV from vaccinated animals”.

## Data Availability

Data are contained within the article and supplementary table.

## Supplementary Materials

The following are available online at https://www.mdpi.com/article/10.3390/v13101980/s1, Table S1. CSFV infection status and comparative ELISA results of the samples employed for validation of the FlagDIVA assay.
