# Supplementary material for: Development of a Dendrimeric Peptide-Based Approach for the Differentiation of Animals Vaccinated with FlagT4G against Classical Swine Fever from Infected Pigs
Source: Viruses. 2021 Oct 2;13(10):1980. doi: 10.3390/v13101980 (PMC8540558; doi:10.3390/v13101980)
Supplement: Supplementary file 1 [file viruses-13-01980-s001.zip › viruses-1394997-supplementary.pdf]

Table S1. CSFV infection status and comparative ELISA results of the samples employed for validation of the FlagDIVA assay

Sample ID	CSFV Infection strain (Genotype)	Days after infection	FlagDIVA ELISA result	Optical density value (FlagDIVA)	E2-antibody ELISA result	Blocking %
1	Non-infected	No	Negative	0,33	Negative	0,00
2	Catalonia01 (2,3)	8	Negative	0,19	Negative	15,23
3	Pinar del Rio (1,4)	21	Positive	0,49	Negative	26,21
4	Catalonia01 (2,3)	3	Negative	0,09	Negative	5,86
5	Catalonia01 (2,3)	14	Negative	0,25	Negative	15,19
6	Catalonia01 (2,3)	17	Negative	0,30	Negative	29,55
7	Non-infected	No	Negative	0,18	Negative	0,00
8	Catalonia01 (2,3)	8	Negative	0,29	Negative	18,30
9	Catalonia01 (2,3)	3	Negative	0,20	Negative	14,87
10	Catalonia01 (2,3)	8	Negative	0,25	Negative	22,46
11	Catalonia01 (2,3)	3	Negative	0,23	Negative	14,73
12	Catalonia01 (2,3)	17	Negative	0,31	Negative	29,07
13	Non-infected	No	Negative	0,16	Negative	0,00
14	Catalonia01 (2,3)	8	Negative	0,14	Negative	15,60
15	Catalonia01 (2,3)	3	Negative	0,09	Negative	12,58
16	Margarita (1,4)	10	Negative	0,39	Negative	0,00
17	Non-infected	No	Negative	0,32	Negative	0,00
18	Margarita (1,4)	18	Negative	0,21	Negative	18,80
19	Catalonia01 (2,3)	3	Negative	0,13	Negative	10,16
20	Catalonia01 (2,3)	8	Negative	0,22	Negative	24,15
21	Margarita (1,4)	10	Positive	0,51	Negative	0,00
22	Non-infected	No	Negative	0,15	Negative	0,00
23	Catalonia01 (2,3)	3	Negative	0,17	Negative	4,80
24	Catalonia01 (2,3)	8	Negative	0,17	Negative	14,23
25	Non-infected	No	Negative	0,19	Negative	0,00
26	Non-infected	No	Negative	0,14	Negative	0,00
27	Margarita (1,4)	13	Negative	0,17	Negative	0,00
28	Catalonia01 (2,3)	8	Negative	0,21	Negative	11,02
29	Catalonia01 (2,3)	3	Negative	0,16	Negative	0,00
30	Catalonia01 (2,3)	14	Negative	0,17	Negative	22,06
31	Non-infected	No	Negative	0,15	Negative	0,00
32	Non-infected	No	Negative	0,20	Negative	0,00
33	Margarita (1,4)	13	Negative	0,14	Negative	0,00
34	Pinar del Rio (1,4)	21	Positive	0,49	Negative	23,66
35	Non-infected	No	Negative	0,12	Negative	0,00
36	Margarita (1,4)	13	Negative	0,28	Negative	0,00
37	Pinar del Rio (1,4)	21	Negative	0,36	Negative	11,78
38	Non-infected	No	Negative	0,13	Negative	0,00
39	Non-infected	No	Negative	0,22	Negative	0,00
40	Non-infected	No	Negative	0,15	Negative	0,00
41	Non-infected	No	Negative	0,10	Negative	0,00
42	Margarita (1,4)	6	Positive	0,69	Negative	0,00
43	Non-infected	No	Negative	0,16	Negative	0,00
44	Non-infected	No	Negative	0,22	Negative	0,00
45	Non-infected	No	Negative	0,30	Negative	7,40
46	Non-infected	No	Negative	0,13	Negative	0,00

Sample ID	CSFV Infection strain (Genotype)	Days after infection	FlagDIVA ELISA result	Optical density value (FlagDIVA)	E2-antibody ELISA result	Blocking %
47	Margarita (1,4)	6	Positive	0,58	Negative	0,00
48	Margarita (1,4)	10	Negative	0,13	Negative	0,00
49	Non-infected	No	Negative	0,38	Negative	-0,46
50	Non-infected	No	Negative	0,13	Negative	0,00
51	Margarita (1,4)	10	Negative	0,19	Negative	0,00
52	Margarita (1,4)	6	Positive	0,66	Negative	0,00
53	Non-infected	No	Negative	0,13	Negative	-10,93
54	Non-infected	No	Negative	0,10	Negative	0,00
55	Margarita (1,4)	10	Negative	0,22	Negative	0,00
56	Margarita (1,4)	6	Positive	0,61	Negative	0,00
57	Non-infected	No	Negative	0,31	Negative	-10,71
58	Non-infected	No	Negative	0,14	Negative	0,00
59	Margarita (1,4)	13	Positive	1,66	Negative	6,43
60	Non-infected	No	Negative	0,17	Negative	5,81
61	Non-infected	No	Negative	0,07	Negative	0,00
62	Non-infected	No	Negative	0,33	Negative	0,00
63	Non-infected	No	Negative	0,25	Negative	0,00
64	Non-infected	No	Negative	0,31	Negative	0,00
65	Non-infected	No	Negative	0,29	Negative	0,00
66	Non-infected	No	Negative	0,09	Negative	-14,40
67	Non-infected	No	Negative	0,10	Negative	-12,09
68	Margarita (1,4)	5	Negative	0,09	Negative	29,65
69	Margarita (1,4)	5	Negative	0,19	Negative	-16,72
70	Non-infected	No	Negative	0,18	Negative	-7,67
71	Margarita (1,4)	5	Negative	0,16	Negative	-13,69
72	Non-infected	No	Negative	0,05	Negative	-5,52
73	Margarita (1,4)	5	Negative	0,07	Negative	-11,87
74	Pinar del Rio (1,4)	21	Negative	0,28	Negative	6,21
75	Non-infected	No	Negative	0,19	Negative	0,00
76	Non-infected	No	Negative	0,13	Negative	-7,89
77	Pinar del Rio (1,4)	15	Negative	0,18	Negative	29,44
78	Margarita (1,4)	5	Negative	0,13	Negative	-16,50
79	Margarita (1,4)	5	Negative	0,19	Negative	11,25
80	Non-infected	No	Negative	0,12	Negative	0,00
81	Pinar del Rio (1,4)	15	Negative	0,14	Negative	29,64
82	Margarita (1,4)	5	Negative	0,08	Negative	-9,33
83	Non-infected	No	Negative	0,29	Negative	0,00
84	Pinar del Rio (1,4)	15	Negative	0,14	Negative	8,29
85	Pinar del Rio (1,4)	22	Negative	0,26	Negative	23,38
86	Pinar del Rio (1,4)	21	Positive	0,69	Negative	23,38
87	Non-infected	No	Negative	0,21	Negative	0,00
88	Margarita (1,4)	13	Negative	0,24	Negative	0,00
89	Pinar del Rio (1,4)	15	Negative	0,22	Negative	20,03
90	Pinar del Rio (1,4)	22	Negative	0,29	Negative	39,32
91	Pinar del Rio (1,4)	21	Positive	0,65	Negative	39,31
92	Margarita (1,4)	13	Negative	0,25	Negative	0,00
93	Pinar del Rio (1,4)	15	Negative	0,16	Negative	6,43
94	Pinar del Rio (1,4)	22	Negative	0,37	Negative	32,27
95	Pinar del Rio (1,4)	21	Positive	0,73	Negative	32,27

Sample ID	CSFV Infection strain (Genotype)	Days after infection	FlagDIVA ELISA result	Optical density value (FlagDIVA)	E2-antibody ELISA result	Blocking %
96	Pinar del Rio (1,4)	15	Negative	0,28	Negative	22,50
97	Non-infected	No	Negative	0,17	Negative	0,00
98	Pinar del Rio (1,4)	15	Negative	0,16	Negative	9,14
99	Margarita (1,4)	13	Negative	0,16	Negative	0,00
100	Non-infected	No	Negative	0,29	Negative	0,00
101	Pinar del Rio (1,4)	15	Negative	0,14	Negative	19,29
102	Pinar del Rio (1,4)	15	Negative	0,15	Negative	24,33
103	Margarita (1,4)	3	Positive	0,73	Negative	0,00
104	Non-infected	No	Negative	0,21	Negative	0,00
105	Non-infected	No	Negative	0,12	Negative	0,00
106	Non-infected	No	Negative	0,22	Negative	0,00
107	Non-infected	No	Negative	0,24	Negative	0,00
108	Non-infected	No	Negative	0,14	Negative	0,00
109	Non-infected	No	Negative	0,26	Negative	0,00
110	Non-infected	No	Negative	0,27	Negative	0,00
111	Margarita (1,4)	8	Positive	0,51	Negative	14,90
112	Margarita (1,4)	8	Positive	0,41	Negative	16,40
113	Pinar del Rio (1,4)	18	Negative	0,40	Negative	24,74
114	Catalonia01 (2,3)	14	Negative	0,27	Doubtful	39,84
115	Pinar del Rio (1,4)	21	Positive	0,59	Doubtful	34,50
116	Catalonia01 (2,3)	17	Negative	0,25	Doubtful	36,38
117	Pinar del Rio (1,4)	15	Negative	0,19	Doubtful	33,81
118	Margarita (1,4)	13	Positive	0,95	Positive	73,00
119	Margarita (1,4)	10	Positive	0,43	Positive	88,00
120	Margarita (1,4)	6	Positive	0,57	Positive	62,00
121	Pinar del Rio (1,4)	21	Positive	0,42	Positive	51,57
122	Catalonia01 (2,3)	14	Positive	0,44	Positive	61,32
123	Margarita (1,4)	10	Positive	1,23	Positive	92,00
124	Margarita (1,4)	6	Negative	0,38	Positive	91,00
125	Catalonia01 (2,3)	17	Positive	0,48	Positive	64,73
126	Margarita (1,4)	13	Positive	1,15	Positive	94,00
127	Pinar del Rio (1,4)	21	Positive	0,46	Positive	45,39
128	Margarita (1,4)	6	Positive	0,50	Positive	79,04
129	Margarita (1,4)	10	Positive	0,51	Positive	92,00
130	Margarita (1,4)	31	Positive	1,10	Positive	49,67
131	Margarita (1,4)	13	Positive	1,32	Positive	92,00
132	Margarita (1,4)	6	Positive	0,42	Positive	77,00
133	Margarita (1,4)	31	Positive	1,20	Positive	57,59
134	Catalonia01 (2,3)	17	Negative	0,29	Positive	62,18
135	Margarita (1,4)	10	Negative	0,27	Positive	93,80
136	Catalonia01 (2,3)	14	Negative	0,16	Positive	41,47
137	Pinar del Rio (1,4)	21	Positive	0,53	Positive	69,00
138	Margarita (1,4)	6	Positive	1,68	Positive	83,00
139	Margarita (1,4)	13	Positive	2,16	Positive	97,00
140	Margarita (1,4)	10	Positive	1,60	Positive	89,80
141	Catalonia01 (2,3)	17	Negative	0,25	Positive	71,84
142	Catalonia01 (2,3)	14	Negative	0,17	Positive	55,17
143	Pinar del Rio (1,4)	21	Positive	0,55	Positive	80,42
144	Margarita (1,4)	13	Positive	1,95	Positive	96,00

Sample ID	CSFV Infection strain (Genotype)	Days after infection	FlagDIVA ELISA result	Optical density value (FlagDIVA)	E2-antibody ELISA result	Blocking %
145	Margarita (1,4)	6	Positive	1,62	Positive	92,00
146	Margarita (1,4)	10	Positive	1,52	Positive	94,20
147	Catalonia01 (2,3)	14	Negative	0,21	Positive	51,75
148	Catalonia01 (2,3)	17	Positive	0,46	Positive	57,33
149	Margarita (1,4)	13	Positive	1,17	Positive	93,55
150	Margarita (1,4)	6	Positive	0,91	Positive	74,26
151	Margarita (1,4)	10	Negative	0,38	Positive	91,40
152	Margarita (1,4)	13	Positive	1,24	Positive	94,70
153	Margarita (1,4)	6	Positive	0,95	Positive	84,00
154	Margarita (1,4)	10	Positive	0,95	Positive	94,30
155	Pinar del Rio (1,4)	21	Positive	0,42	Positive	46,91
156	Margarita (1,4)	31	Positive	1,06	Positive	54,40
157	Margarita (1,4)	12	Negative	0,39	Positive	45,70
158	Margarita (1,4)	13	Positive	1,23	Positive	94,62
159	Margarita (1,4)	6	Positive	0,52	Positive	89,00
160	Margarita (1,4)	10	Positive	0,80	Positive	93,90
161	Pinar del Rio (1,4)	21	Positive	0,47	Positive	66,40
162	Margarita (1,4)	13	Positive	1,26	Positive	94,33
163	Margarita (1,4)	10	Positive	0,96	Positive	94,60
164	Margarita (1,4)	13	Positive	0,72	Positive	43,00
165	C-strain (1,1)	26	Negative	0,18	Positive	50,22
166	Margarita (1,4)	12	Positive	0,45	Positive	45,86
167	Margarita (1,4)	13	Positive	0,66	Positive	76,79
168	Margarita (1,4)	13	Positive	0,49	Positive	65,00
169	Margarita (1,4)	13	Negative	0,24	Positive	49,40
170	Margarita (1,4)	31	Positive	0,89	Positive	42,46
171	Margarita (1,4)	10	Negative	0,20	Positive	66,00
172	Margarita (1,4)	13	Positive	0,55	Positive	85,50
173	Margarita (1,4)	10	Negative	0,27	Positive	91,00
174	Margarita (1,4)	5	Positive	0,59	Positive	58,94
175	Margarita (1,4)	10	Positive	0,42	Positive	94,00
176	Margarita (1,4)	10	Negative	0,37	Positive	76,00
177	Margarita (1,4)	10	Positive	0,48	Positive	93,20
178	Margarita (1,4)	10	Negative	0,38	Positive	68,00
179	Thiverval	20	Negative	0,32	Positive	46,00
180	Margarita (1,4)	10	Negative	0,32	Positive	65,00
181	Margarita (1,4)	5	Positive	0,75	Positive	82,51
182	Margarita (1,4)	13	Positive	0,61	Positive	72,00
183	Margarita (1,4)	13	Positive	0,15	Positive	42,00
184	Margarita (1,4)	13	Positive	0,66	Positive	80,00
185	Margarita (1,4)	13	Positive	0,74	Positive	74,00
186	Margarita (1,4)	13	Positive	0,86	Positive	58,00
187	Pinar del Rio (1,4)	21	Positive	1,56	Positive	47,46
188	Margarita (1,4)	13	Positive	1,00	Positive	72,00
189	Margarita (1,4)	6	Positive	0,41	Positive	66,00
190	Pinar del Rio (1,4)	22	Positive	0,77	Positive	47,46
191	Pinar del Rio (1,4)	21	Positive	1,73	Positive	40,41
192	Margarita (1,4)	13	Positive	1,37	Positive	70,00
193	Margarita (1,4)	6	Positive	0,54	Positive	50,00

Sample ID	CSFV Infection strain (Genotype)	Days after infection	FlagDIVA ELISA result	Optical density value (FlagDIVA)	E2-antibody ELISA result	Blocking %
194	Pinar del Rio (1,4)	22	Positive	0,86	Positive	40,41
195	Margarita (1,4)	13	Negative	0,16	Positive	58,56
196	Pinar del Rio (1,4)	21	Positive	1,60	Positive	42,79
197	Pinar del Rio (1,4)	22	Positive	0,97	Positive	42,79
198	Pinar del Rio (1,4)	22	Positive	0,89	Positive	44,98
199	Pinar del Rio (1,4)	21	Positive	1,67	Positive	44,98
200	Pinar del Rio (1,4)	22	Positive	0,64	Positive	42,54
201	Pinar del Rio (1,4)	22	Positive	0,62	Positive	58,09
202	Margarita (1,4)	6	Positive	0,42	Positive	85,00
203	Margarita (1,4)	6	Positive	0,56	Positive	94,00
204	Margarita (1,4)	3	Positive	0,59	Positive	45,00
205	Margarita (1,4)	3	Positive	0,66	Positive	43,00
206	Margarita (1,4)	6	Negative	0,29	Positive	89,00
207	Margarita (1,4)	3	Positive	0,66	Positive	67,00
208	Margarita (1,4)	6	Negative	0,36	Positive	89,00
209	Margarita (1,4)	3	Positive	1,03	Positive	53,00
210	Margarita (1,4)	6	Positive	0,42	Positive	93,00
211	Pestivirus	Unknown	Positive	1,04	Positive	Unknown
212	Pestivirus	Unknown	Positive	2,00	Positive	Unknown
213	Margarita (1,4)	17	Positive	0,66	Positive	67,40
214	Margarita (1,4)	17	Positive	0,62	Positive	78,00
215	Pinar del Rio (1,4)	18	Negative	0,37	Positive	41,99
216	Catalonia01 (2,3)	28	Positive	0,94	Positive	79,19
217	Catalonia01 (2,3)	Unknown	Positive	0,44	Positive	61,24
